# A novel mutation in the FGD4 gene causing Charcot‐Marie‐Tooth disease

**DOI:** 10.1111/jns.12222

**Published:** 2017-09-05

**Authors:** Panagiotis Zis, Mary M. Reilly, Dasappaiah G. Rao, Pedro Tomaselli, Alex M. Rossor, Marios Hadjivassiliou

**Affiliations:** ^1^ Academic Department of Neurosciences Sheffield Teaching Hospitals NHS Foundation Trust; ^2^ University of Sheffield; ^3^ MRC Centre for Neuromuscular Diseases UCL Institute of Neurology, Queen Square London UK

**Keywords:** CMT, FGD4 gene, mutation, neuropathy

## Introduction

Demyelinating forms of Charcot‐Marie‐Tooth (CMT) result from mutations in a number of genes, the majority of which show an autosomal dominant pattern of inheritance *(Bird,* 1993‐2016 *)*. Recessive patterns of inheritance are less common. We report a case of demyelinating CMT resulting from compound heterozygous mutation in the FGD4 gene. This report is consistent with the ethical guidelines of the publisher and written informed consent was obtained from the patient for the publication of this report.

## Clinical Presentation

The patient was a 61‐year‐old Caucasian woman with progressive unsteadiness since the age of 36.. At age 35, she was diagnosed as having Friedreich's ataxia. She had not been followed‐up since then. The patient recalled having dexterity difficulties and frequent falls since the age of 8. She had multiple foot surgeries. She had a normal birth and normal milestones.

There was no relevant family history. The patient had two sisters, who both died of cancer. The patient reported that her mother had clawed toes but walked normally in her 90s. Past medical history included arterial hypertension and a hip replacement at the age of 52. The patient was smoking and was not drinking alcohol excessively.

Examination revealed kyphoscoliosis. Neurological examination showed normal cranial nerves, apart from broken pursuit eye movements. There was no nystagmus. There was mild finger to nose ataxia and more prominent heel to shin and gait ataxia. She could walk using bilateral support. She had mild pes cavus and deformed feet. There was bilateral foot drop. Her right leg was weaker compared with the left, following the hip replacement. She had distal weakness (MRC 4) in both hands. She was areflexic.

She had severe sensory loss (pinprick and proprioception) in both arms up to the elbows and both legs up to the knees. Vibration sensation was absent in the limbs. Romberg's sign was positive.

Routine blood tests, vitamin B12, folate and immunology testing were normal or negative. She had increased thyroglobulin and thyroid peroxidase antibodies, but thyroid function testing was normal. Magnetic resonance imaging (MRI) of the brain was unremarkable.

Nerve conduction studies showed absent sensory responses. Motor conduction studies showed reduced amplitudes (0.5–1.2 mV) and severely reduced velocities (8–14 m/sec).

Genetic screening for the chromosome 17 duplication and for mutations in PMP22, MPZ, NEFL, GDAP1, GJB1, EGR2 and the common mitochondrial mutations (MELAS, MERRF and NARP) were all negative. Next generation sequencing analysis of a panel of 14 genes (GJB1, EGR2, FGD4, FIG4, GADP1, LITAF, MPZ, MTMR2, NDRG1, NEFL, PMP22, PRX, SBF2, SH3TC2) implicated in CMT1 revealed two heterozygous likely pathogenic variants in *FGD4*: c.[1192‐48_1233del];[1304_1305delinsAA] p.{(?);(Arg435Gln)]. Neither mutation has been previously reported on public normal variant databases (dbSNP, NHLBI, exome variant server and ExAC). The c.1192‐48_1233del p.(?) mutation is a 90 bp deletion encompassing the intron 9/exon 10 boundary and is predicted to result in a truncated and/or frameshifted transcript and a loss of function. The c.1394_1305delinsAA p.(Arg435Gln) missense mutation occurs at a highly conserved amino acid within the plekstrin homology domain, a domain in which a pathogenic recessive missense mutation has previously been reported (p.Arg442His PMID 22734899). As this missense mutation resides within the 90 bp deletion in the first mutation, we were able to confirm autosomal recessive inheritance by Sanger sequencing showing that these two mutations were present on separate alleles despite not having her parents DNA available.

## Discussion

CMT type 4 is the term commonly used to describe autosomal recessive CMT1 (ARCMT1) cases although some also classify the autosomal recessive CMT2 ARCMT2 cases as CMT4 *(Rossor et al.,* 2016 *)*. CMT type 4H is characterized by an early onset (up to the age of 10 years) demyelinating neuropathy, with slow progression and scoliosis *(Delague,* 1993‐2016 *)*. The diagnosis is established by the presence of biallelic FGD4 pathogenic variants.

In the case described here there was no clear family history of neuropathy. The presence of kyphoscoliosis, however, in combination with the very severe neuropathy as indicated by the nerve conduction studies pointed towards CMT4.

Sensory ataxia when severe can be difficult to distinguish from cerebellar ataxia. Nerve conduction studies in such patients are essential in deciding which gene panels to pursue based on the type and severity of the neuropathy. This case also illustrates the need for up to date review of patients labeled as having a particular genetic disease prior to the availability of genetic testing for confirmation.

## Author contributions

P. Z.: drafting/revising the manuscript, clinical management of the case, accepts responsibility for conduct of research and final approval. M. M. R.: genetic analysis, revising the manuscript. D. G. R.: clinical management of the case, revising the manuscript. P. T.: genetic analysis, revising the manuscript. A. M. R.: genetic analysis, revising the manuscript. M. H.: drafting/revising the manuscript, clinical management of the case, accepts responsibility for conduct of research and final approval.
